# Systematic pathway engineering of *Corynebacterium glutamicum* S9114 for l-ornithine production

**DOI:** 10.1186/s12934-017-0776-8

**Published:** 2017-09-22

**Authors:** Bin Zhang, Miao Yu, Ying Zhou, Yixue Li, Bang-Ce Ye

**Affiliations:** 1 Laboratory of Biosystems and Microanalysis, State Key Laboratory of Bioreactor Engineering, East China University of Science and Technology, Shanghai, 200237 China; 2 Key Laboratory of Systems Biology, Shanghai Institute for Biological Sciences, Chinese Academy of Sciences, Shanghai, 200031 China

**Keywords:** *Corynebacterium glutamicum*, l-Ornithine production, Metabolic engineering

## Abstract

**Background:**

l-Ornithine is a non-protein amino acid with extensive applications in medicine and the food industry. Currently, l-ornithine production is based on microbial fermentation, and few microbes are used for producing l-ornithine owing to unsatisfactory production titer.

**Results:**

In this study, *Corynebacterium glutamicum* S9114, a high glutamate-producing strain, was developed for l-ornithine production by pathway engineering. First, *argF* was deleted to block l-ornithine to citrulline conversion. To improve l-ornithine production, *ncgl1221* encoding glutamate transporter, *argR* encoding arginine repressor, and *putP* encoding proline transporter were disrupted. This base strain was further engineered by attenuating oxoglutarate dehydrogenase to increase l-ornithine production. Plasmid-based overexpression of *argCJBD* operon and lysine/arginine transport protein LysE was tested to strengthen l-ornithine synthesis and transportation. This resulted in efficient l-ornithine production at a titer of 18.4 g/L.

**Conclusion:**

These results demonstrate the potential of *Corynebacterium glutamicum* S9114 for efficient l-ornithine production and provide new targets for strain development.

**Electronic supplementary material:**

The online version of this article (doi:10.1186/s12934-017-0776-8) contains supplementary material, which is available to authorized users.

## Background

*Corynebacterium glutamicum*, an aerobic gram-positive actinomycete capable of secreting glutamate under biotin limitation or in the presence of penicillin, has long-term applications in the industrial production of various valuable metabolites, including amino acids besides glutamate such as arginine, lysine, ornithine, and alanine [1–4]; diamines such as putrescine [5, 6] and cadaverine [7]; dicarboxylic acids such as succinate [8–10]; diols such as 1,3-propanediol [11] and 1,2-propanediol [12]; and terpenes such as pinene and carotenoid [13]. *Corynebacterium glutamicum* S9114, a subspecies of *C. glutamicum* derived from *Brevibacterium tianjinese* T6–13 [14], has been widely used for glutamate fermentation in China. Its genome sequence was published in 2011, highlighting its importance in glutamate fermentation. Compared to other strains of *C. glutamicum*, strain S9114 shows high glutamate production (about 80 g/L), resistance to high sugar levels, and much faster growth, and can be referred to as an ideal host for producing glutamate-related compounds [15].

l-Ornithine, a non-protein amino acid, is universally used in treating liver disease and trauma; it plays an efficient role in liver protection [16] and is useful in strengthening the heart [17]. l-Ornithine is an important constituent of the urea cycle and can be further converted to l-citrulline and l-arginine. In this pathway, l-glutamate is converted to l-ornithine by ArgC, ArgJ, ArgB, and ArgD, which are expressed as an operon and are regulated by the arginine repressor, ArgR. Recently, several reports have described the progress in the development of microorganisms used for l-ornithine production. As a potential industrial strain, *C. glutamicum* was proven to be a superior l-ornithine producer. Jiang et al. [18] reported an engineered *C. glutamicum* ATCC 13032 derived strain with inactivated *argF*, *proB*, and *speE*, and overexpression of NADH-dependent glutamate dehydrogenase from *Bacillus subtilis*, which produced 14.8 g/L of l-ornithine in a shake flask. In another study from the same group, 24.1 g/L of l-ornithine was obtained by adaptive evolution of *C. glutamicum* [19]. In addition, Hwang and Cho [20] reported a recombinant strain with inactivation of three putative glucose dehydrogenase genes to increase the NADPH supplement, which improved l-ornithine production to 14 g/L. Jensen et al. also constructed a mutant strain with feedback alleviation of *N*-acetylglutamate kinase, lowering expression of *pgi*, tuning of the promoter of *gdh*, which improved l-ornithine production of 71% as compared to the parental Δ*argFRG* strain [21]. Kim et al. [22] reported the construction of a recombinant *C. glutamicum* by disrupting *proB*, *argR* and *argF*, and overexpressing the operon of *argCJDB* from *C. glutamicum* ATCC 21831. Fed-batch culture of the engineered strain in a 6.6-L fermenter yielded 51.5 g/L of l-ornithine production titer from glucose.

**Fig. 1:**
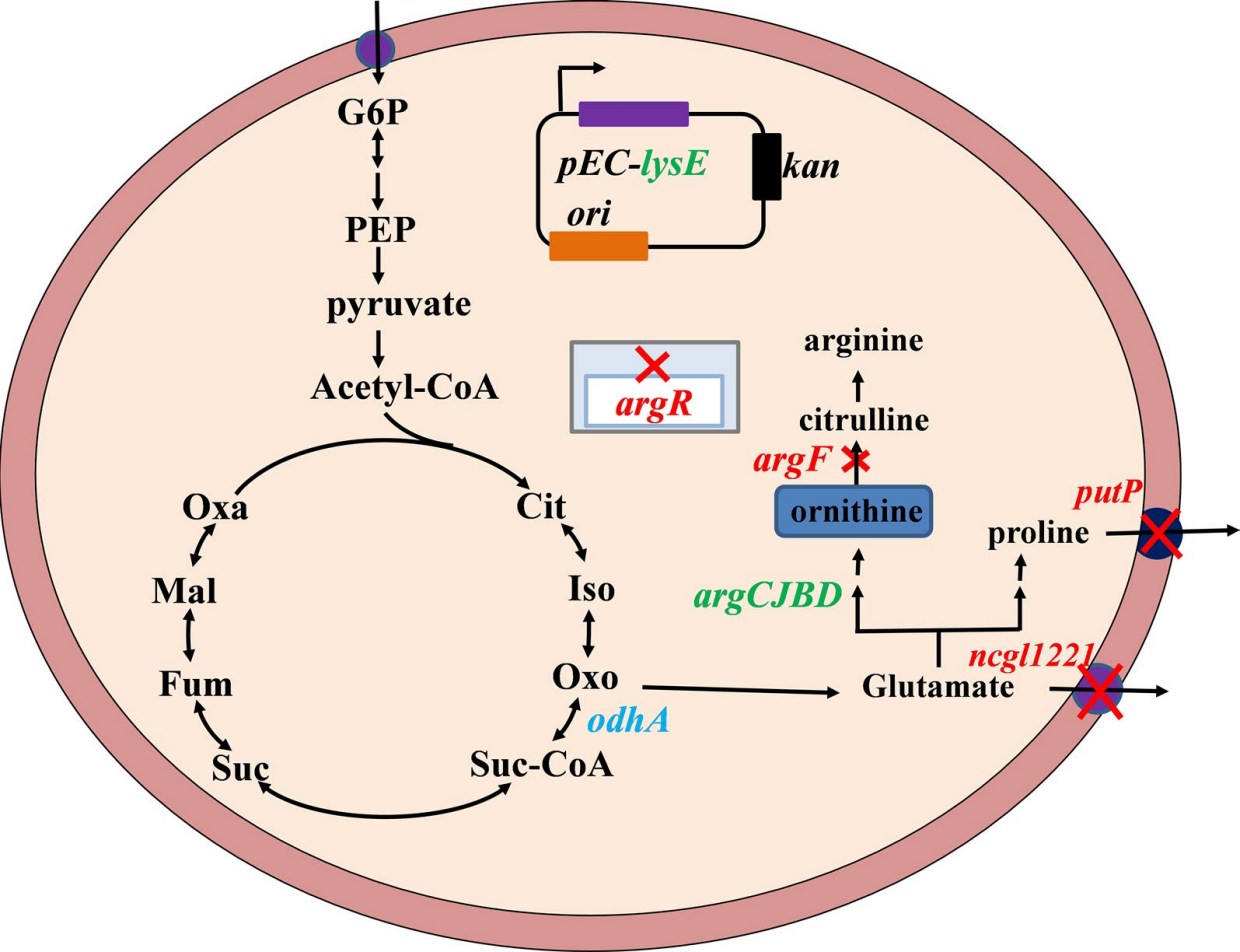
Metabolic pathway abbreviated drawing for l-ornithine biosynthesis in *C. glutamicum*. Glc, glucose; G6P, glucose-6-P; PEP, phosphoenolpyruvate; Qxa, oxaloacetate; Mal, malate; Fum, fumarate; Suc, succiate; Suc-CoA, succinyl-coA; Oxo, 2-oxoglutarate; Iso, isocitrate; Cit, citrate; *argF*, encoding ornithine carbamoyltransferase; *odhA*, a subunit of ketoglutarate dehydrogenase; *argCJBD*, an operon involved in arginine synthesis; *argR*, a repressor for *argCJBD*; *ncgl1221*, encoding glutamate transporter; *lysE*, encoding lysine/arginine transporter; *putP*, encoding l-proline transporter. The red fonts represent gene deletion, green font means gene overexpression, and blue font means gene attenuation

In this study, we systematically developed a new strain, *C. glutamicum* S9114, for l-ornithine production (Fig. 1). Compared with the model stain *C. glutamicum* ATCC 13032, the engineered *C. glutamicum* S9114 produced more l-ornithine after system pathway modification including inactivation of ArgF, Ncgl1221, ArgR, and PutP, attenuation of OdhA, and overexpression of LysE. This is the first report to explore the deletion of Ncgl1221 and PutP, along with attenuation of OdhA and overexpression of LysE as efficient strategies for improving l-ornithine production in *C. glutamicum*. Among these, deletion of Ncgl1221, attenuation of OdhA, and overexpression of LysE were confirmed to promote l-ornithine production, which provides crucial guidance for exploiting and developing industrial l-ornithine-producing strains.

## Methods

### Strains, medium, and culture conditions

The microorganisms and recombinant plasmids used in this study are presented in Table 1. *E. coli* DH5α was used as the clone host for construction of the recombinant plasmid. *Corynebacterium glutamicum* S9114 was used as the original strain for the development of mutants. Luria–Bertani (LB) medium was used to propagate *E. coli* and *C. glutamicum*. For the l-ornithine fermentation assay, a single clone of the mutants was activated on LB agar plate for two cycles of 12 h. Subsequently, a ring of bacteria was inoculated into 10 mL of seed medium in a 100-mL normal shake flask. The seed medium consisted of (per liter) 25 g glucose, 10 g yeast extract, 10 g corn steep liquor, 15 g (NH_4_)_2_SO_4_, 2.5 g MgSO_4_·7H_2_O, 1 g KH_2_PO_4_, 0.5 g K_2_HPO_4_, 0.5 g Na_2_HPO_4_, and 10 g CaCO_3_. After 11 h of cultivation at 32 °C and 220 rpm, the appropriate amount of culture was transferred to 24 mL of fermentation medium in a 250-mL baffle shake flask. Initial OD_600_ of the fermentation culture was adjusted to one. The fermentation medium consisted of (per liter) 100.0 g glucose, 20.0 g corn steep liquor, 50.0 g (NH_4_)_2_SO_4_, 2.5 g MgSO_4_·7H_2_O, 1.0 g KH_2_PO_4_, 0.5 g K_2_HPO_4_, 0.5 g Na_2_HPO_4_, 0.02 g FeSO_4_·7H_2_O, 0.02 g MnSO_4_·4H_2_O, and 10 g CaCO_3_. The initial pH was adjusted to 7.0. All cultures were grown at 32 °C and 250 rpm, and 200-µL samples were collected every 12 h to measure l-ornithine concentration, cell density, and residual glucose concentration. If necessary, 50 mg/L kanamycin was used to cultivate *E. coli* and 12.5 mg/L kanamycin was used to cultivate *C. glutamicum*.

**Table 1:** Strains and plasmids used in this study

Strain/plasmid	Characteristic	Source
*Strain*		
*E. coli* DH5ɑ	Clone host strain	Transgen
*C. glutamicum* CICC 20190	An l-arginine producing strain, ATCC 21493	CICC
*C. crenatum* MT-M4	An l-arginine producing strain, derived from *C. crenatum* AS 1.542.	[23]
*C. glutamicum* S9114	Industrial strain for glutamate production	[14]
Sorn1	*C. glutamicum* S9114 with *argF* deletion	This study
Sorn2	Sorn1 with *ncgl1221* deletion	This study
Sorn3	Sorn2 with *argR* deletion	This study
Sorn4	Sorn3 with *putP* deletion	This study
Sorn5	Sorn4 with RBS200 and A1G change in *odhA*	This study
Sorn6	Sorn4 with RBS400 and A1G change in *odhA*	This study
Sorn7	Sorn4 with RBS800 and A1G change in *odhA*	This study
Sorn8	Sorn7 with pEC-XK99E	This study
Sorn9	Sorn7 with pEC-*argCJBD1*	This study
Sorn10	Sorn7 with pEC-*argCJBD2*	This study
Sorn11	Sorn7 with pEC-*lysE*	This study
*Plasmid*		
pK18*mobsacB*	Mobilizable vector, allows for selection of double crossover in *C. glutamicum*, Km^R^, *sacB*	[35]
pEC-XK99E	A shuttle expression vector, Km^R^	Lab stock
pK18-Δ*argF*	A derivative of pK18*mobsacB*, harboring Δ*argF* fragment	This study
pK18-Δ*ncgl1221*	A derivative of pK18*mobsacB*, harboring Δ*ncgl1221* fragment	This study
pK18-Δ*argR*	A derivative of pK18*mobsacB*, harboring Δ*argR* fragment	This study
pK18-Δ*putP*	A derivative of pK18*mobsacB*, harboring Δ*putP* fragment	This study
pK18-*odhA200*	A derivative of pK18*mobsacB*, harboring *odhA* of 217 au RBS change and A1G fragment	This study
pK18-*odhA400*	A derivative of pK18*mobsacB*, harboring *odhA* of 373 au RBS change and A1G fragment	This study
pK18-*odhA800*	A derivative of pK18*mobsacB*, harboring *odhA* of 837 au RBS change and A1G fragment	This study
pEC-*argCJBD1*	A derivative of pEC-XK99E, harboring *argCJBD* gene from *C. glutamicum* CICC 20190 under its native promoter	This study
pEC-*argCJBD2*	A derivative of pEC-XK99E, harboring *argCJBD* gene from *C. crenatum* MT-M4 under its native promoter	This study
pEC-*lysE*	A derivative of pEC-XK99E, harboring *lysE* gene from *C. glutamicum* S9114 under its native promoter	This study

Superscript ‘‘R’’ indicates resistance to the following antibiotics: Km kanamycin

### Plasmid construction and gene knockout

The *C. glutamicum* S9114 derived mutant strains and plasmids constructed in this work are listed in Table 1. Genomic DNA of *C. glutamicum* S9114 isolated according to a previous report was used as the template for the amplification of specific genes. The suicide vector pK18*mobsacB* with the sucrose screening marker *sacB* from *Bacillus subtilis* was used for markerless gene deletion, insertion, and ribosome-binding site (RBS) replacement by double crossover recombination, as described previously [23]. The *E. coli*/*C. glutamicum* shuttle expression vector, pEC-XK99E, was used for overexpressing *argCJBD*.

For gene deletion in *C. glutamicum* S9114, the upstream and downstream fragments (approximately1000 bp) of *argF*, *ncgl1221*, *argR*, and *putP* were amplified and fused by PCR. Subsequently, the overlapped fragments were inserted into the *Hin*dIII/*Xba*I site of pK18*mobsacB* by Gibson assembly, thus generating the recombinant plasmids pK18-∆*argF*, pK18-∆*ncgl1221*, pK18-∆*argR*, and pK18-∆*putP*. In addition, for chromosome RBS change of OdhA, the RBS sequence listed in Additional file 1: Table S2 with the gradient translation start strength designed by the RBS calculator (https://www.denovodna.com/software/doLogin) was inserted into the overlap region between the upstream and downstream fragments of *odhA*, using rationally designed primers. After standardized molecular cloning operations, the plasmids pK18-*odhA200*, pK18-*odhA400*, and pK18-*odhA800* were constructed. Moreover, *argCJBD* along with its native promoter was amplified from the genomic DNA of *C. glutamicum* CICC 20190 and *C. crenatum* MT-M4, and then inserted into expression plasmid pEC-XK99E via a *Bam*HI/*Sal*I site, thus generating the plasmids pEC-*argCJBD1* and pEC-*argCJBD2*. All recombinant plasmids were transformed into *C. glutamicum* S9114 and the derived engineered strains by electroporation. Correct mutants obtained after two rounds of homologous recombination were confirmed by colony PCR. All the primers used in this study are listed in Additional file 1: Table S1.

### Measurement of ODHC specific activity

For ODHC specific activity determination, cells were collected at 12 h, which is late exponential phase. Next, disposition of samples and enzymatic reaction system were conducted according to a previously described method with minor modification [24–26]. According to the previous work, cells collected by centrifugation (at 5000 rpm, 4 °C, and 10 min) and washed twice with 0.2% KCl solution. Following the pure cells were incubated in 5 mL of 0.1 M *N*-tris(hydroxymethyl)methyl-2-aminoethanesulfonic acid (TES)·NaOH buffer (pH 7.7) containing 30% (v/v) glycerol and 10 mg/mL lysozyme at 37 °C for 3 h and then disrupted by sonication. After removing cell debris by centrifugation, the supernatant was collected as crude enzyme, and the protein concentration was determined by bicinchoninic acid (BCA) assay using bovine serum albumin as the standard. ODHC specific activity assay was performed by adding 8 μL of crude enzyme to 200 μL of reaction mixture. The reaction mixture contained 100 mM TES·NaOH buffer (pH 7.7), 3 mM cysteine, 5 mM MgCl_2_, 0.2 mM coenzyme A, 0.3 mM thiamine pyrophosphate and 1 mM 3-acetylpyridine adenine dinucleotide (APAD^+^). The reaction was initiated by adding 1 mM α-oxoglutarate to the mixture, and then the absorbance of APADH at 365 nm was consecutively measured at 31.5 °C for 5 min with 30 s intervals. ODHC specific activity is defined as the amount of enzyme required to generate 1 µmol APADH per minute.

### Analytical procedures

Cell growth was monitored by measuring the OD_600_ using a microplate reader (BioTek Instruments, Winooski, VT, USA) after dissolving CaCO_3_ by 0.125 mol/L HCl. The fermentation supernatant was processed using a 0.22-µm filter and analyzed for glucose, glutamate, and lactate levels, using a SBA-40C biosensor (developed by Biology Institute of Shandong Academy of Sciences). l-Ornithine concentrations were determined by colorimetry using ninhydrin, as described previously [18].

## Results

### Deletion of *argF* results in efficient accumulation of l-ornithine

**Fig. 2:**
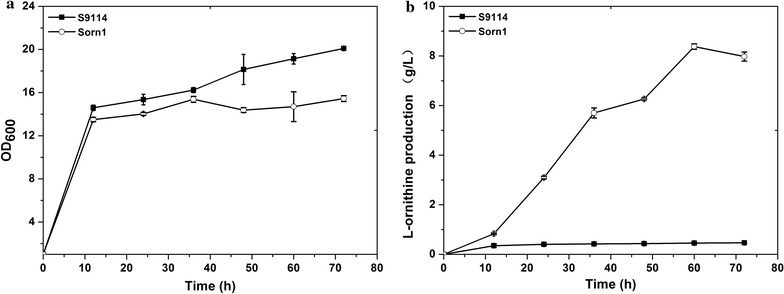
Influence of deleting *argF* on cell growth and l-ornithine production during shake-flask cultivations. **a** The growth of strain S9114 and Sorn1. **b** l-Ornithine production. Results of standard deviations present in three individual experiments

**Fig. 3:**
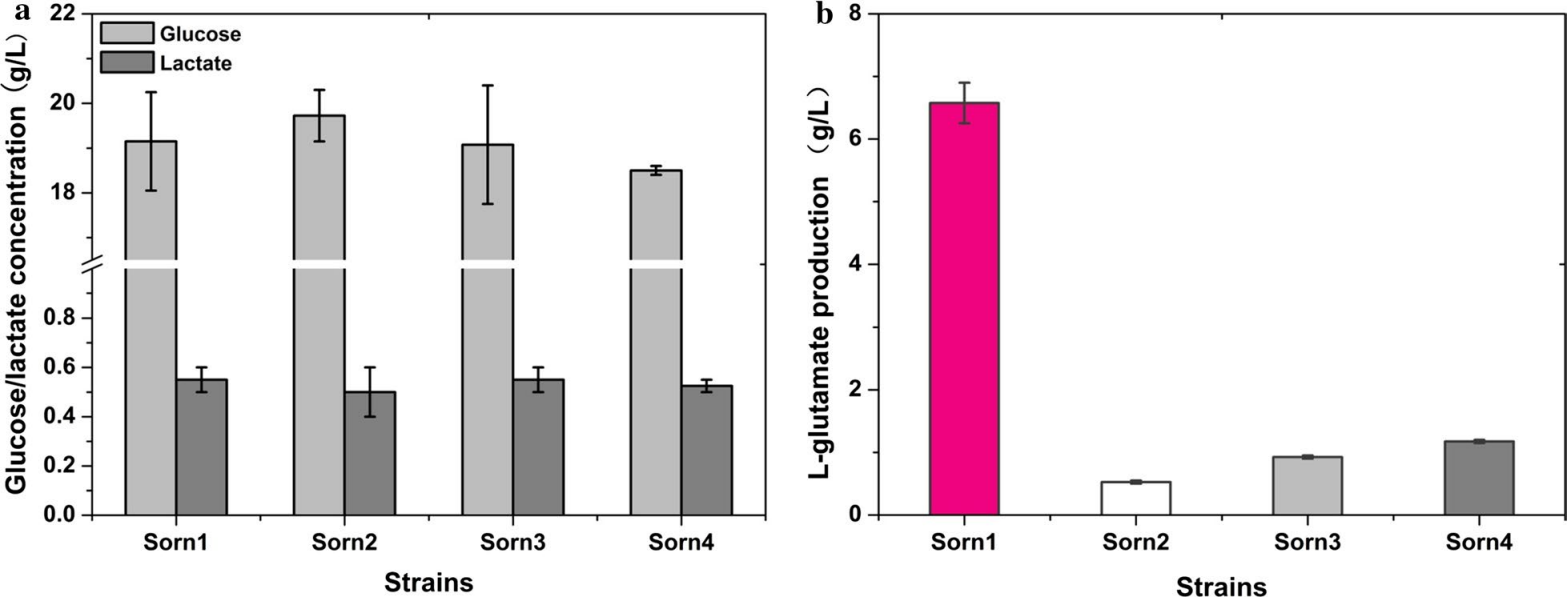
The residual glucose, glutamate and lactate concentration in fermentation broth of Sorn1, Sorn2, Sorn3 and Sorn4. **a** Glucose and lactate concentration; **b** l-Glutamate concentration. Results of standard deviations present in three individual experiments

*Corynebacterium glutamicum* S9114 as an industrial strain was reported to accumulate large amounts of glutamate under appropriate fermentation conditions. Because l-ornithine is an intermediate metabolite of l-arginine synthesis derived from l-glutamate, *C. glutamicum* S9114 was considered a favorable host for the development of l-ornithine-producing strains. As shown in Fig. 1, l-ornithine can be converted to citrulline by carbamoyltransferase, which is encoded by *argF* in *C. glutamicum*. Therefore, *argF* was disrupted to accumulate l-ornithine in *C. glutamicum* S9114, resulting in strain Sorn1. As shown in Fig. 2, analysis of samples from the shake flask fermentation assay indicates that although growth was reduced by the deprived l-arginine synthesis pathway, the mutant strain Sorn1 produced 7.97 g/L of l-ornithine at 72 h, which is 16-fold higher than that produced by *C. glutamicum* S9114 (0.46 g/L). Moreover, our data also indicated that Sorn1 produced 6.5 g/L of glutamate in the fermentation supernatant (see Fig. 3). In conclusion, inactivation of ArgF resulted in a high l-ornithine production titer in *C. glutamicum* S9114.

### Improvement of l-ornithine production by reducing glutamate transportation

**Fig. 4:**
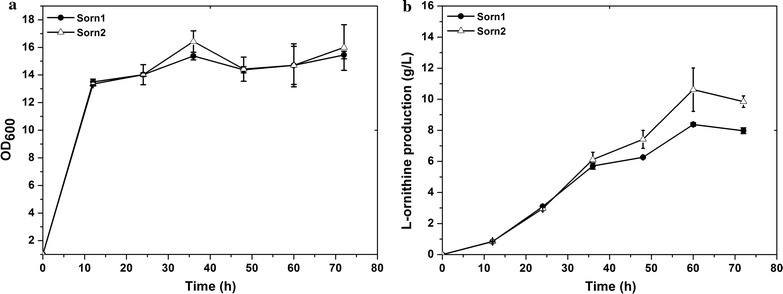
Effect of *ncgl1221* deletion on l-ornithine production and cell growth during shake-flask cultivations. **a** The growth of Sorn1 and Sorn2 (Sorn1 with *ncgl1221* deletion); **b** l-ornithine curves with temporal change. Results of standard deviations present in three individual experiments

Glutamate is the precursor of l-ornithine synthesis and therefore, extracellular secretion of glutamate is unfavorable attribute to the problems of downstream separation and wastage of materials. According to a previous report, deleting *ncgl1221*, which encodes a glutamate transport protein, caused intracellular accumulation of l-glutamate by damaging the transportation system and showed no influence on growth [26]. Thus, *ncgl1221* was disrupted to reduce glutamate secretion and provide more precursors for l-ornithine production in strain Sorn1, thus generating the strain Sorn2. The results from the shake flask test shown in Fig. 4 suggest that 9.8 g/L of l-ornithine was produced by stain Sorn2 after 72 h of cultivation, which was 22.7% higher than that obtained with strain Sorn1. The growth, glucose consumption, and lactate production of Sorn2 were consistent with those of Sorn1 (Fig. 3). These preliminary results demonstrate an efficient strategy to improve l-ornithine production by deleting *ncgl1221*. In addition, the glutamate concentration in the fermentation supernatant of Sorn2 was decreased to 0.5 g/L, which was just about 7.6% of that in the control strain Sorn1. Consequently, glutamate production was reduced by a titer of 6 g/L whereas l-ornithine production was only increased by a titer of 1.83 g/L, which illustrated that further enhancement of l-ornithine production was limited by the conversion efficiency of glutamate to ornithine in strain Sorn2.

### Optimization of l-ornithine production by inactivation of *argR* and *putP*

**Fig. 5:**
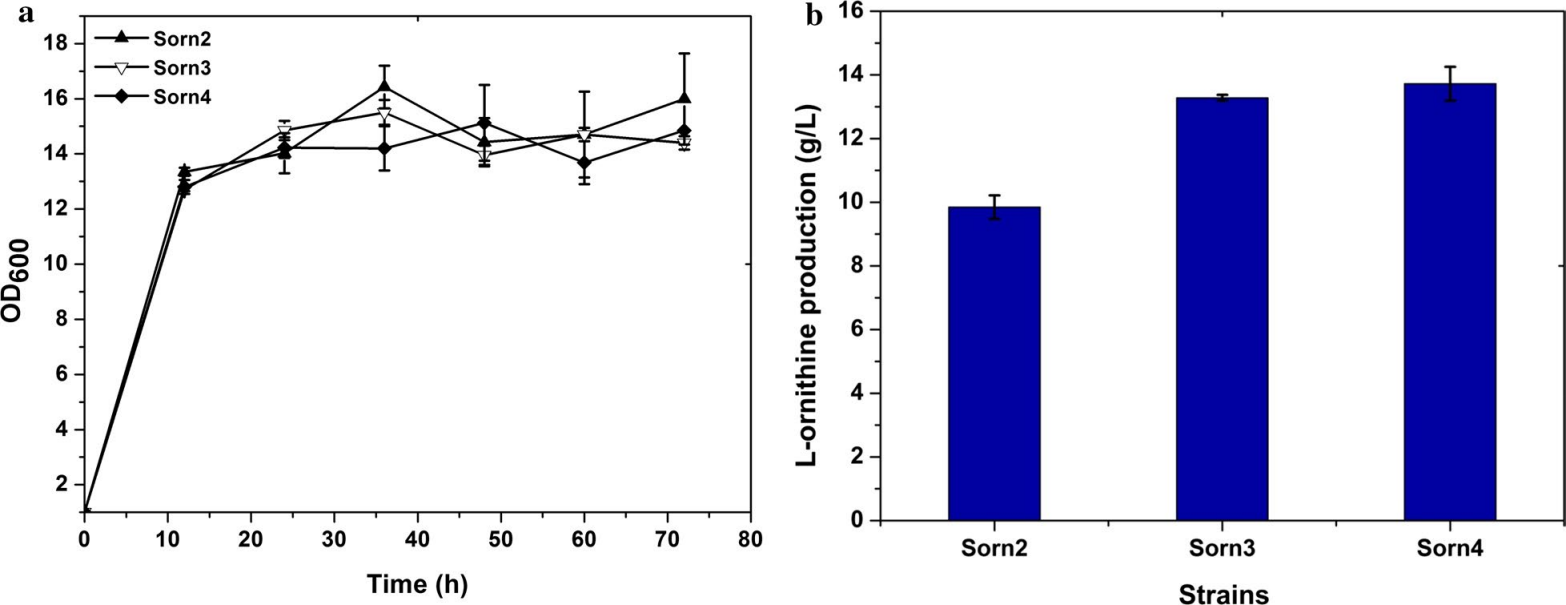
Effect of *argR* and *putP* deletion on l-ornithine production in Sorn2. **a** The growth of Sorn2, Sorn3 (Sorn2 with *argR* deletion) and Sorn4 (Sorn3 with *putP* deletion). **b** l-Ornithine concentration in fermentation supernatant. Results of standard deviations present in three individual experiments

l-Ornithine is synthesized via a four-step enzyme reaction and the genes encoding these enzymes are expressed as a gene cluster, *argCJBD*. Deletion of *argR*, an arginine negative regulatory protein, can significantly increase the expression level of the *argCJBDFR* gene cluster [27]. Therefore, to enhance the metabolic flux from l-glutamate to l-ornithine, *argR* was deleted to remove the feedback repression in Sorn2, thus generating the mutant strain, Sorn3. According to Fig. 5, the l-ornithine production titer of Sorn3 in the shake flask reached 13.2 g/L after 72 h of incubation, which was 34.6% higher than that of Sorn2. In addition, the growth, glucose consumption, and lactate production was not affected by deletion of *argR*. However, l-proline was considered a competing metabolic by-product owing to the consumption of glutamate as a common precursor. For further saving carbon metabolism, *putP* that encodes l-proline transport protein, was inactivated to reduce l-proline synthesis by biological robustness, and generate the strain Sorn4. The l-ornithine production titer of Sorn4 was equal to that of Sorn3, indicating that l-proline production in Sorn3 has no significant impact on the production of l-ornithine.

### Attenuation of *odhA* exerts a positive effect on l-ornithine production

l-Ornithine is a derivative product of glutamate, which suggests that increased metabolic flux from 2-oxoglutarate to glutamate is a reasonable strategy for enhancing l-ornithine production. Therefore, *odhA* was selected as a target for attenuation in the strain Sorn4 via the RBS modification method. The RBS strength of the original *odhA* in the chromosome of Sorn4 as predicted by the RBS Calculator (https://www.denovodna.com/software/doLogin) was 1613 au. Afterwards, three kinds of RBS with predicted translation initiation intensity of 217, 373 and 837 au were designed and used to replace the natural RBS of the strain Sorn4 and the start codon ATG of *odhA* was simultaneously replaced with GTG, thus generating strains Sorn5, Sorn6, and Sorn7. The results of the shake flask test illustrated that without influencing growth, the recombinant strains Sorn5, Sorn6, Sorn7 produced 14.1, 14.2, and 16 g/L of l-ornithine at 72 h, respectively (see in Table 2). The production titer of Sorn7 was 16.7% higher than that of the control strain Sorn4. Compared with the parent strain Sorn4, the relative ODHC specific activity of strains Sorn5, Sorn6, Sorn7 reduced to 15, 22 and 40% (see in Table 2). This result illustrated that attenuation of *odhA* promotes l-ornithine production in *C. glutamicum* S9114.

**Table 2:** Engineering the l-ornithine production in recombinant *C. glutamicum*

Strains	Cell biomass (OD_600_)	l-Ornithine accumulation (g/L)	l-Ornithine/cell biomass (OD_600_)	Relative ODHC specific activity
Sorn4	15.90 ± 0.10	13.74 ± 0.68	0.86	1.00 ± 0.07
Sorn5	15.65 ± 0.35	14.11 ± 1.33	0.90	0.15 ± 0.01
Sorn6	15.30 ± 0.50	14.23 ± 0.65	0.93	0.22 ± 0.02
Sorn7	16.05 ± 0.15	16.04 ± 0.42	1.00	0.40 ± 0.02
Sorn8	15.12 ± 0.01	15.17 ± 0.86	1.00	–
Sorn9	14.97 ± 0.15	15.17 ± 0.70	1.01	–
Sorn10	15.09 ± 0.33	15.29 ± 0.50	1.01	–

Fermentations were performed at 250 rpm for 72 h, and the initial glucose concentration was 100 g/L. Results are the means ± standard deviations in three individual experiments

### Heterologous expression of *argCJBD* causes no further improvement in l-ornithine production

To examine whether the expression level of *argCJBD* is a rate-limiting step for further enhancing l-ornithine production, the *argCJBD* operon under its native promoter from two l-arginine producing strains was amplified and introduced into the mutant strain Sorn7, via the expression plasmid pEC-XK99E, thus generating strains Sorn9 and Sorn10. Shake flask fermentation was performed to evaluate the effect of these modifications on l-ornithine production. As illustrated in Table 2, strains Sorn9 and Sorn10 produced 15.1 and 15.28 g/L of l-ornithine respectively, which is the same titer as 15.1 g/L obtained with Sorn8. The growth of those strains was also not changed. This result suggests that after eliminating the feedback control by inactivation of *argR*, expression of *argCJBD* is not a rate-limiting step for l-ornithine production.

### Overexpression of LysE to enhance l-ornithine production

**Fig. 6:**
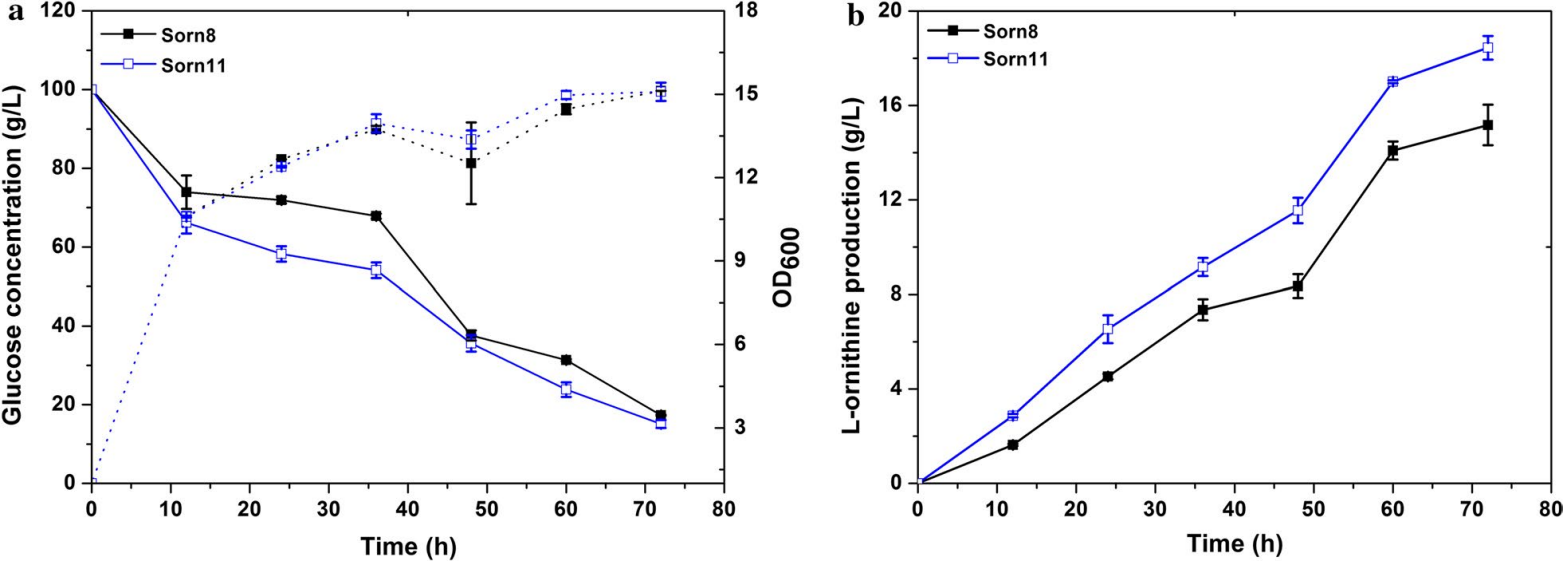
Effect of *lysE* overexpression on l-ornithine production. **a** The glucose concentration and growth curve of Sorn8 and Sorn11. The solid line represents the glucose concentration curve; the dotted line represents growth curve. **b** l-Ornithine production curves of Sorn8 and Sorn11. Results of standard deviations present in three individual experiments

LysE, a broad amino acid transporter, possessed the ability to transport l-lysine, l-arginine and l-citrulline [28]. Hence, to investigate whether overexpression of LysE could further improve l-ornithine production based on the strain Sorn7, we constructed a mutant strain Sorn11. As shown in Fig. 6, the Sorn11 strain consumed 74.9 ± 1 g/L glucose and produced 18.4 ± 0.49 g/L l-ornithine with a yield of 0.25 g/g glucose. These data represent an increase of 21.8% in l-ornithine production as compared to that of the strain Sorn8. However, compared with the strain Sorn8, Sorn11 did not show any obvious differences in cell growth, but its glucose consumption was slightly increased. It was thus concluded that overexpression of LysE caused further improved l-ornithine production in Sorn7. The result obtained in this section demonstrates the remarkable application of LysE in constructing an l-ornithine-producing strain.

## Discussion

*Corynebacterium glutamicum* S9114, an industrial glutamate producer, was systematically engineered for l-ornithine production for the first time. Inactivation of *argF*, encoding a carbamoyltransferase that converts ornithine to citrulline in *C. glutamicum* S9114, led to l-ornithine production of 7.97 g/L, which is 2.74-fold higher than the 2.13 g/L obtained by disrupting *argF* in *C. glutamicum* ATCC 13032 [29]. Compared with the model strain *C. glutamicum* ATCC 13032, *C. glutamicum* S9114 possesses a tremendous advantage in the precursor supplement. To improve l-ornithine production, *ncgl1221* encoding the known transport protein of glutamate was also disrupted to avoid glutamate as a by-product secreted to the medium, which was reported to effectively improve the production titer of l-arginine in *C. glutamicum* [30]. To our knowledge, this is the first study confirming that elimination of *ncgl1221* significantly contributes to l-ornithine synthesis. We speculated that deletion of *ncgl1221* leads to intracellular accumulation of glutamate and then stimulates the downstream metabolic pathways to improve l-ornithine production. A second step to improve l-ornithine yield was to remove the feedback control by inactivating *argR*, which was reported to effectively improve the transcription level of the arginine operon. The result of increased l-ornithine production by ArgR inactivation was consistent with that of a previous study, which revealed that disrupting *argR* improved the transcription of arginine operon [27] and enhanced l-arginine and l-ornithine production in *C. glutamicum* [31]. However, deletion of *putP* did not exert a positive effect on l-ornithine production, which suggested that production of l-proline was controlled by other unknown mechanisms and did not compete for the precursor glutamate.

Decreased specific activity of 2-oxoglutarate dehydrogenase was proven as an important target for glutamate [25] and arginine production [32]. In this study, we attempted to decrease the expression of *odhA* to enhance the metabolic flux from TCA cycle to l-ornithine by RBS modification and initiation codon replacement. The highest yield of l-ornithine was observed for strain Sorn7 with RBS of 837 au in upstream region of *odhA*. This is consistent with previous result obtained in engineered *C. crenatum* possessing gene *odhA* with RBS of 800 au [32]. Two strains with weaker RBS of 217 au or 373 au in upstream region of *odhA* displayed slow growth, and producing the lower amount of l-ornithine. Based on this, we continued to strengthen the l-ornithine synthesis pathway by overexpressing the *argCJBD* operon from two arginine-producing strains. However, this was not consistent with previous research which revealed that overexpression of *argCJBD* genes from *C. glutamicum* ATCC 21831 in the model strain *C. glutamicum* ATCC 13032 significantly increased l-ornithine production [22]. The different strains may account for these diverse outcomes. It is speculated that the expression of *argCJBD* is not the rate-limiting step for l-ornithine synthesis after deletion of the arginine repressor ArgR. There may be other reasons that limit the further improvement of l-ornithine production.

LysE, a lysine transporter [33], is also reported to transport l-arginine [34]. In previous work, overexpression of LysE in *C. glutamicum* could significantly increase l-arginine and l-citrulline yield [28]. In this study, overexpression of LysE under its native promoter in the mutant strain Sorn7 (with *argF*, *ncgl1221*, *argR*, *putP*, and *odhA800* modifications) contributed to a 21.8% increase in l-ornithine production compared with that by the base strain. These data demonstrate the significant effect of LysE on l-ornithine production. Moreover, overexpression of LysE also significantly promotes the utilization of glucose, which was illustrated for the first time. Some correlations may thus exist between improvement of glucose consumption and l-ornithine production. However, Bellmann [34] reported that LysE is not able to transport l-ornithine in *C. glutamicum* ATCC 13032 in short time fermentation. We speculated that the difference of species or other unknown mechanism except enhancing transport system might contribute to those controversial results. In our next project, further experiments such as gene deletion and anaplerosis experiments were needed to explore the insight of why LysE present those effects.

While further improvement and optimization of metabolic pathways as well as process engineering remain to be investigated, this work demonstrates the enormous potential of *C. glutamicum* S9114 to produce l-ornithine from glucose. As l-ornithine is an intermediate of l-citrulline and l-arginine biosynthesis, successful overproduction of this compound by pathway engineering indicates that this organism has superduper potential to overproduce not only l-ornithine but also l-citrulline, l-arginine and other glutamate relative compound.

## Conclusions

In this study, we achieved the goal of development of a high glutamate-producing strain, *Corynebacterium glutamicum* S9114, for l-ornithine production by deletion of *argF*, *ncgl1221*, *argR*, *putP*, attenuating oxoglutarate dehydrogenase and overexpression of LysE, which produced 18.4 g/L of l-ornithine. The development of metabolically engineered *C. glutamicum* S9114 strains provides a new strain and some useful strategies for enhanced fermentative production of l-ornithine from renewable resources such as glucose. Through further genetic engineering and fermentation condition optimization, the yield of l-ornithine is capable for further improvement.

## Additional file

**Additional file 1: Table S1.** Primers and their sequences in this study. **Table S2.** RBS sequence for attenuation of *odhA.*
