# Supplementary material for: Systematic pathway engineering of Corynebacterium glutamicum S9114 for l-ornithine production
Source: Microb Cell Fact. 2017 Sep 22;16:158. doi: 10.1186/s12934-017-0776-8 (PMC5610420; doi:10.1186/s12934-017-0776-8)
Supplement: Supplementary file 1 — Additional file 1: Table S1. Primers and their sequences in this study. Table S2. RBS sequence for attenuation of odhA. [file 12934_2017_776_MOESM1_ESM.docx]

TABLE S1 Primers and their sequences in this study

| Primers | Sequence (5’-3’) | Restriction enzyme |
| --- | --- | --- |
| *argF*-up-F | cggtacccggggatcctctagCAAACGAGGCTGCTTTCAAGAT |  |
| *argF*-up-R | GTC**GTCGAC**GCGTCTTTATGCGATTGTCTCGGCAAT | *Sal* I |
| *argF*-down-F | TCGCATAAAGACGC**GTCGAC**GACTAAGACATGTCCCTTGGCTCAAC | *Sal* I |
| *argF-*down-R | aacgacggccagtgccaagctCTTGATGGTTGGCAGGCAGTAC |  |
| *ncgl1221*-up-F | cggtacccggggatcctctagCAAGAAAGCCCTCGTTCCAACACTG |  |
| *ncgl1221*-up-R | GCGTCCTAAC**GTCGAC**GGAATCAAAAACGCCAAGACCAGG | *Sal* I |
| *ncgl1221*-down-F | GTTTTTGATTCC**GTCGAC**GTTAGGACGCTGATTACAGACG | *Sal* I |
| *ncgl1221*-down-R | aacgacggccagtgccaagctGGAAGGGAGTTGAAGGTGACG |  |
| *argR*-up-F | cggtacccggggatcctctagCTACGCACACAGCAATTTCCAC |  |
| *argR*-up-R | GTC**GTCGAC**GCGTCCTTCATCCAGCAGCAATTCAG | *Sal* I |
| *argR*-down-F | CTGGATGAAGGACGC**GTCGAC**GACGACACCGTTTTCGTTCTC | *Sal* I |
| *argR*-down-R | aacgacggccagtgccaagctCAGAGCTGGATCCTCGGTGTAT |  |
| *putP*-up-F | cggtacccggggatcctctagCTTCTGGCGTGGGAAGATGAC |  |
| *putP*-up-R | CTT**GTCGAC**GCACCGAGATATCCGCGATTTCGCTGACGCAGCTAAG | *Sal* I |
| *putP*-down-F | GCGGATATCTCGGTGC**GTCGAC**AAGCAAAATCTTG | *Sal* I |
| *putP*-down-R | aacgacggccagtgccaagctTTTCCACTGATTCAGCGCATG |  |
| *odhA*-up-F | aacgacggccagtgccaagcttTTCGACTCCTACCAACAAGCC |  |
| *odhA200*-up-R | TTTTTTCTACTGACCCTTTTAGTAGAACGTGTCAGGCCATTAAATGC |  |
| *odhA200*-down-F | CTACTAAAAGGGTCAGTAGAAAAAATACCTCCCTAGTGCTACAACTGGGGCTTAGG |  |
| *odhA*-200-F | CTACTAAAAGGGTCAGTAGAAAAAA |  |
| *odhA400*-up-R | ATTATTGTGTTAATCTCTATGATTTGCAACGTGTCAGGCCATTAAATGC |  |
| *odhA400*-down-F | GCAAATCATAGAGATTAACACAATAATAAATTCCCGTGCTACAACTGGGGCTTAGG |  |
| *odhA*-400-F | GCAAATCATAGAGATTAACACAATAAT |  |
| *odhA800*-up-R | AAATTAATAATATCGACGGGGTGAACGTGTCAGGCCATTAAATGC |  |
| *odhA800*-down-F | CACCCCGTCGATATTATTAATTTAGGGCTCAGAAGTGCTACAACTGGGGCTTAGG |  |
| *odhA*-800-F | CACCCCGTCGATATTATTAATTT |  |
| *argCJBD*-F | ttcgagctcggtacccggggatccCGGACACGCTGGCTACTAAAAAT | *BamH* I |
| *argCJBD* -R | aagcttgcatgcctgcaggtcgacATAAGTTTGAGTCCTTTATGCGAT | *Sal* I |
| *lysE*-F | caggtcgactctagaggatccAAGGCCACCGATTCTAAAACC | *BamH* I |
| *lysE*-R | gccaaaacagccaagctgaattcTTGGGTGCGCGATACCAATAC | *EcoR* I |

Restriction enzyme sites were represented as bold characters. The overlapping region with PCR fragment were marked by underline. The overlapping region with vector was represented as lowercase. The start codon of *odhA* were marked by break line.

TABLE S2 RBS sequence for attenuation of *odhA*

| Name | RBS sequence (5’-3’) | Predicted translation strart strength |
| --- | --- | --- |
| *odhA200* | CTACTAAAAGGGTCAGTAGAAAAAATACCTCCCTAGTG | 217.06 au |
| *odhA400* | GCAAATCATAGAGATTAACACAATAATAAATTCCCGTG | 373.38 au |
| *odhA800* | CACCCCGTCGATATTATTAATTTAGGGCTCAGAAGTG | 837.39 au |

The start codon of *odhA* were marked by underline.
